# Unusual Maxillary First Molars with C-Shaped Morphology on the Same Patient: Variation in Root Canal Anatomy

**DOI:** 10.1155/2019/1857289

**Published:** 2019-10-22

**Authors:** Naji Kharouf, Youssef Haïkel, Davide Mancino

**Affiliations:** ^1^Inserm UMR_S 1121, Biomaterials and Bioengineering, Strasbourg University, 11 Rue Humann, 67085 Strasbourg, France; ^2^Faculty of Dental Medicine, Department of Endodontics, Strasbourg University, 8 Rue Sainte Elisabeth 67000 Strasbourg, France

## Abstract

A maxillary first molar should be considered a four-canal tooth until proved otherwise; however, a clinician should also be aware of the possibility of the presence of C-shaped root canal configuration with or without possibility of splitting into two or three canals. The two clinical cases reported in this paper describe the endodontic treatment of two maxillary first molars, on the same patient, with uncommon anatomy: the first case is about a maxillary first molar with only one C-shaped root and one oval canal with a large buccolingual diameter, a C1 type according to Fan's classification; the second case, about the contralateral maxillary first molar, is probably the first case documented of a maxillary first molar with a C-shaped root canal and C-shaped root with complete fusion of the three roots, having a C3 configuration.

## 1. Introduction

Human maxillary first molars are usually considered as three-rooted teeth with four root canals for the presence of a second canal in the MB root (MB2). In addition, lateral ramifications and apical delta of the root canal system may frequently occur, increasing the probability of leaving untreated spaces after the root canal therapy [1].

Back in 1925, Hess pointed out the complexity of the root canal system of maxillary molars [2]. Later on, many articles have been published concerning the canal configurations of the maxillary first molars and of maxillary molars in general [3, 4].

Probably, a maxillary first molar is the tooth with the wider range of anatomical variations [5, 6]. Many of these anatomical variations are confirmed in literature, and the incidence of four root canals in three roots for the presence of MB2 ranges from 25% to 96.1% [3, 7].

A literature review on 8399 maxillary first molars showed that the MB root had two or more canals in 56.8% of cases [8]. The incidence of three root canals in three roots ranges from 32.14% [3] to 75% [4]. The incidence of two root canals in two roots ranges from 1.85% to 0.3% [9]. Indeed, the incidence of four roots or one root and one canal as well as the existence of C-shaped root morphology and C-shaped root canal is very rare.

According to De Moor [10], the probability of observing a C-shaped canal in a maxillary first molar was as low as 0.091%.

The C-shaped canal system is an anatomic variation occurring mostly in mandibular second molars, especially in Asian populations, although it can also occur in maxillary and other mandibular molars. A tooth is qualified as having a C-shaped root canal if it has the following features: fused roots, a longitudinal groove on the lingual or buccal surfaces of the root, and at least one cross-section of the canal that belongs to the C1, C2, or C3 configuration [11]. A C-shaped root, which always contains a C-shaped canal, results from the failure of Hertwig's epithelial sheath to develop or fuse in the furcation area in the developing stage of the teeth [12].

The C-shaped root morphology should not be confused with the taurodont root morphology, even though both are caused by an inward folding of the tooth wall [12].

The C-shaped morphology constitutes one of the most important anatomic variations without any association with genetic malformation, whereas taurodont root morphology has been found to occur as an isolated trait with familial tendency or as a feature in a wide variety of multiple-system malformation syndromes, and it does not exhibit any cross-section of the canal that belongs to the C1, C2, or C3 configuration.

The two clinical cases reported below describe the endodontic treatment of two maxillary first molars, on the same patient, with uncommon anatomy: the first case is about a maxillary first molar with only one C-shaped root and one oval canal with a large buccolingual diameter, a C1 type according to Fan's classification [13]; the second case is about the contralateral maxillary first molar with a C-shaped root and 3 root canals, a C3 type according to Fan's classification.

## 2. Case Reports

A 13-year-old Caucasian female was referred to the Clinic of Endodontics of Dental School, University of Strasbourg. Her medical history found no outstanding findings that would contribute to treatment problems. The 13-year-old Caucasian female was referred by his general dentist to the Clinic of Endodontics of Dental School, University of Strasbourg, for endodontic treatment on the right and left maxillary first molars, both with symptomatic irreversible pulpitis.

All procedures were done in conformity with current state of the art practices in endodontic. These included effective local anesthesia, rigorous clinical and radiographic preaccess analysis, appropriate tooth restoration to insure watertight rubber dam installation, and surgical microscopic manipulations for precision (Leica M320). The endodontic treatments were performed in one session.

### 2.1. Maxillary Right First Molar

**Figure 1:**
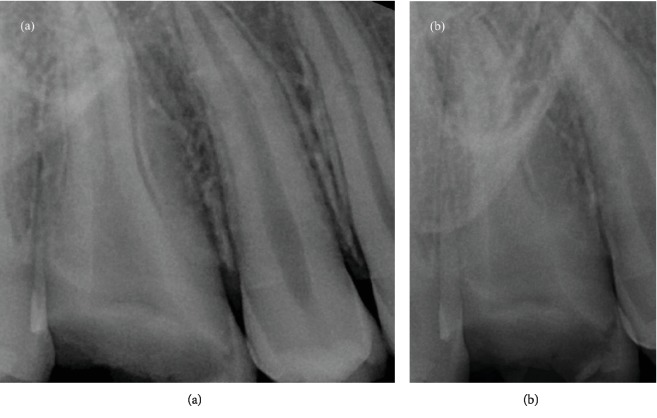
(a) Preoperative orthoradial X-ray; (b) preoperative distal angulated X-ray.

Two preoperative radiographs were taken, with different angles: the first, with an orthoradial projection; the second, a distal angulated projection. They showed the presence of one wide canal and the presence of a deep mesial groove (Figure 1). After a careful study of the preoperative radiographs, the cementoenamel junction (CEJ) was identified by performing a circumferential probing with a periodontal probe. This allowed to have a three-dimensional view of the pulp canal system before starting the treatment [14]. Probing identified the presence of two middle deep grooves in the distal and mesial aspects of the tooth. After the rubber dam placement, the endodontic access cavity preparation was started with a # 012 cylindrical diamond drill and enlarged with a Start X1 ultrasonic tip (Dentsply Sirona, York, USA). The pulp chamber was localized, and the access cavity preparation was refined with the same tip. Only one oval orifice, with a large buncolingual diameter, was localized. Then, keeping the pulp chamber constantly flooded with 6% sodium hypochlorite, root canal shaping was performed, using a step down technique without initial manual scouting [15]:

1. Initial preflaring
2. Apical scouting
3. Glide path
4. Shaping

The initial mechanical preflaring was performed at first with Proglider (Dentsply Sirona) until 2/3 of estimated radiographic working length (WL) applying an in and out movement, using an endodontic engine (300 rpm/5 Ncm). After the initial preflaring, a #25 NiTiflex K-file (Dentsply Sirona) easily scouted the canal until WL + 0.5 mm. Length determination, on the buccal and palatal poles of the root canal, was taken using an electronic apex locator (Root ZX; J Morita Co., Kyoto, Japan).

Then, a mechanical glide path using Proglider at working length was performed. The tooth was shaped, as if it had two canals, buccal and palatal. Root canal shaping was performed by preparing the root canal system until the WL with ProTaper Next X1, X2, and X3 (operating at 300 rpm and torque of 5 N/cm). The foramen, on the buccal pole, was gauged introducing a # 60/02 NiTi hand file, which was snug at working length, using the pecking technique [16]. Indeed, the minor diameter on the palatal pole was gauged introducing a # 70/02 NiTi hand file.

The isthmus between the buccal and palatal poles was shaped using a bended sonofile #15 K-file (Satalec, France).

**Figure 2:**
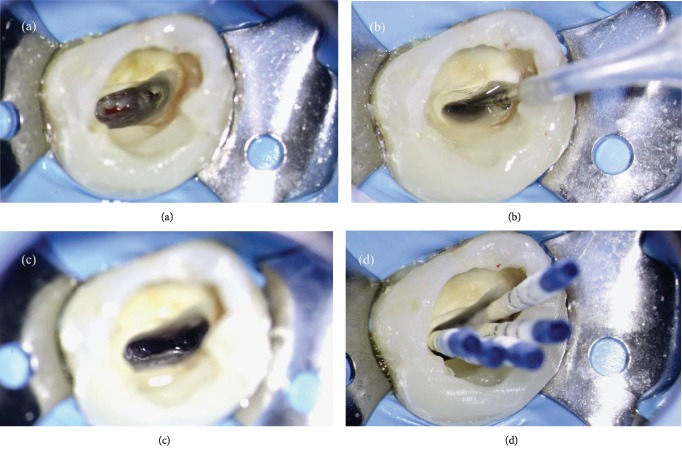
(a) Root canal system at the end of shaping procedure. (b, c) Root canal system during final rinse. (d) Canal drying using five sterile paper points.

After the shaping procedure (Figure 2(a)), in order to assure proper three-dimensional cleaning of the root canal system, the final rinse solutions were activated using EndoUltra (Micro-Mega, Besançon, France): at first, an aqueous solution of EDTA 17% was activated inside the canal for 120 seconds. After rinsing with physiological saline, an aqueous solution of NaOCl 6% was activated inside the canal for 240 seconds (Figures 2(b) and 2(c)). The canal was dried three times using five sterile paper points, arranged in accordance with its palatal-buccal axis (Figure 2(d)).

**Figure 3:**
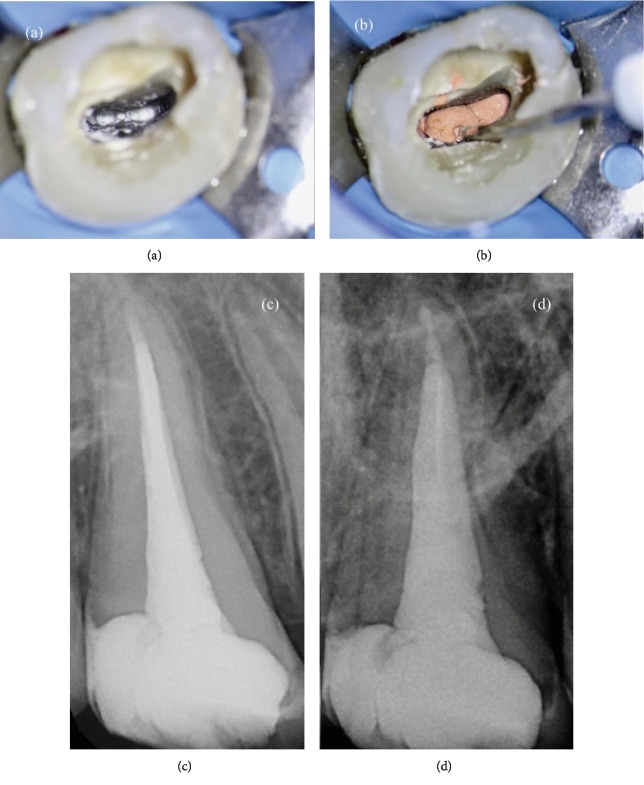
(a) Visualization of the apical plug. (b) Backfilling of the root canal system using thermoplastic gutta-percha. (c) Postoperative orthoradial X-ray. (d) Postoperative angulated X-ray.

In order to perform a tridimensional filling, the tooth was filled using a bioceramic material, MTA Biorep (Itena, Paris, France), creating an apical plug of 4 mm thickness (Figure 3(a)). It was mixed according to the manufacturer's instructions and delivered to the apical portion of the canal using the MAP System (Produits Dentaires, Vevey, Switzerland). The NiTi tip chosen for this case was the yellow 0.90 mm diameter instrument. After the positioning of the MTA Biorep apical plug, the bioceramic material was adapted to the canal walls using at first the yellow NiTi Machtou hand plugger, followed by the red hand plugger. The remaining part of the root canal was filled with thermoplastic gutta-percha, using EQ-V fill (Meta Biomed, Chungcheongbuk-do, Republic of Korea) in association with a canal sealer (Figure 3(b)). The final radiographs showed a well-obturated canal of this single-rooted maxillary first molar, with a large buccopalatal diameter (Figures 3(c) and 3(d)).

### 2.2. Maxillary Left First Molar

**Figure 4:**
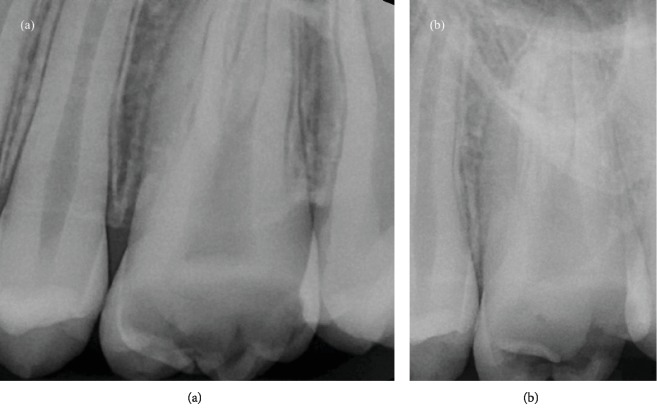
(a) Preoperative orthoradial X-ray; (b) preoperative distal angulated X-ray.

One week later, the contralateral maxillary first molar was treated too. Two preoperative radiographies were taken, with different angulations. The radiographs showed the presence of a deep pulp chamber floor with an unusual anatomy; two canals were clearly detectable (Figure 4). After a careful study of the preoperative radiographs, the cementoenamel junction (CEJ) was identified by performing a circumferential probing with a periodontal probe. The probing identified the presence of one middle deep groove in the buccal aspect of the tooth, a feature of the C-shaped canal system. Then, for the rubber dam placement, the endodontic access cavity preparation was started with a # 012 cylindrical diamond drill and enlarged with a Start X1 (Dentsply Sirona) ultrasonic tip.

**Figure 5:**
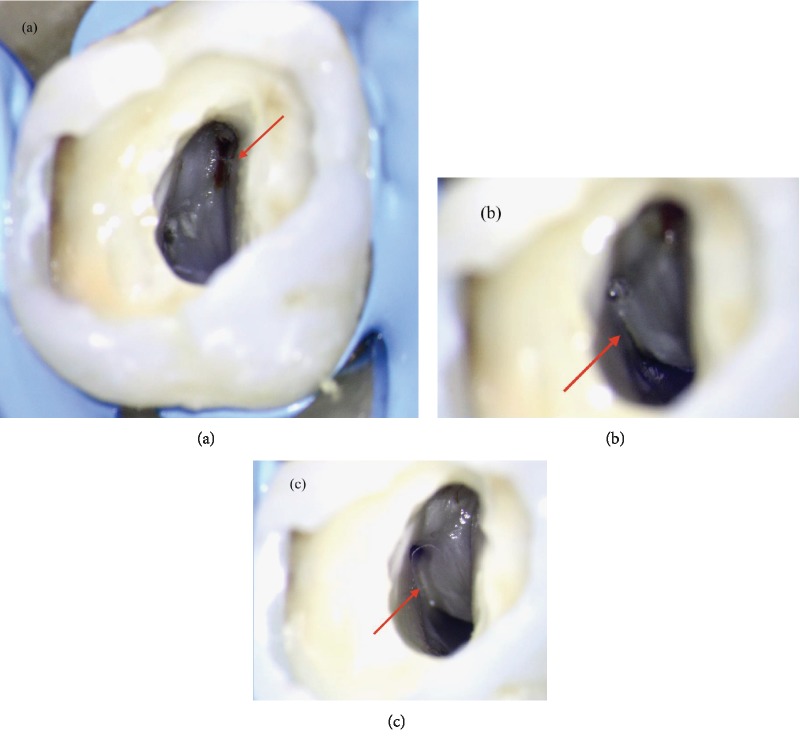
(a) Visualization of the oval orifice of the MB canal (arrow). (b) Distal circular orifice and ribbon palatal orifice separated by a deep isthmus (arrow). (c) Isthmus shaped using sonofile.

An oval MB orifice, a distal circular orifice, and a ribbon palatal orifice were easily localized with the same tip, under microscopic examination. P canal and D canal were separated by a deep isthmus (Figure 5).

The root canals were shaped with the same technique used in the first case, performed in the order: initial preflaring, apical scouting, glide path, and shaping using for the P canal PTX1, PTX2, and PTX 3 until WL and for the MB and D canal PTX1 and ProFile 25/04 until WL. MB and D canal were shaped in a minimally invasive way in order to avoid any risk of stripping. The isthmus was shaped using a bended sonofile #15 K-file.

The cleaning step was managed in the same way as the first case for the D canal, including the isthmus management, whereas for the distal and MB canal, the irrigation solutions were activated using EQ-S (Meta Biomed, Chungcheongbuk-do, Republic of Korea) with a white tip (15/02) at WL-0.5 mm.

Then, the canals were dried using sterile paper points. After having applied a drop of the AH Plus root canal sealer (Dentsply Sirona) with a coated paper point in the entrance of each canal, MB and D canals were filled with Thermafil 25 and P canal with Thermafil 35.

**Figure 6:**
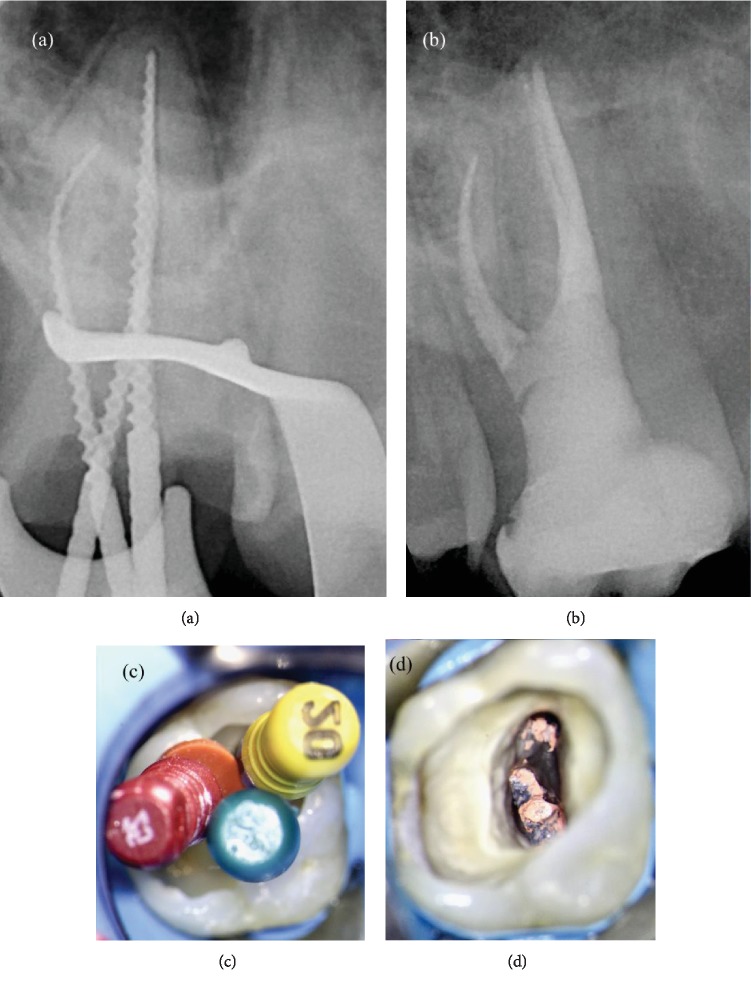
(a) Intraoperative orthoradial X-ray using verifiers. (b) Postoperative angulated X-ray. (c) Intraoperative photo with verifier. (d) Intraoperative photos after Thermafil filling.

The carrier-based technique used in this case should be able to fill in a tridimensional way the D and MB canals shaped with a small taper as well as the isthmus between the P and D canals without any risk of root fracture (Figure 6).

## 3. Discussion

The maxillary first molar is a tooth that presents a high risk of missing canals, and it should be considered a four-canal tooth until proved otherwise. However, the clinician should also be aware of the possibility of the presence of C-shaped root and C-shaped root canal configuration with or without possibility of splitting into two or three canals. A limited number of reports have described the existence of C-shaped canals in maxillary first molars [11, 17–19].

The two clinical cases reported describe the endodontic treatment of two maxillary first molars, on the same patient, with C-shaped morphology.

Coronal anatomy and dimensions of both maxillary first molars were within normal limits and provided no indication of the unusual root and root canal configuration.

But, as these cases show, a painstaking preaccess analysis of the preoperative radiographs, the systematic identification of the CEJ, using a periodontal probe, and the use of a surgical microscope coupled with the use of specific endodontic ultrasonic tips allow a high accuracy of the access cavity preparation and canal localization also in case of C-shaped root canal configuration.

On the one hand, in this case, in accordance with the AAE position statement, conventional intraoral radiography, and not CBCT, was the imaging modality of choice [20], considering the young age of the patient too. On the other hand, it is evident that if an intraoperative CBCT were needed it would be taken using limited FOV.

It is all too evident that in cases of C-shaped root canal configuration, like these maxillary first molars, the shaping, cleaning, and filling phases are more difficult. When present, this type of canal system is essential to plane the shaping and cleaning procedure.

Hence, in a C-shaped root, the first step before starting the shaping procedure is the localization of the groove, where the thickness of the dentin is smaller, in order to avoid any risk of stripping.

**Figure 7:**
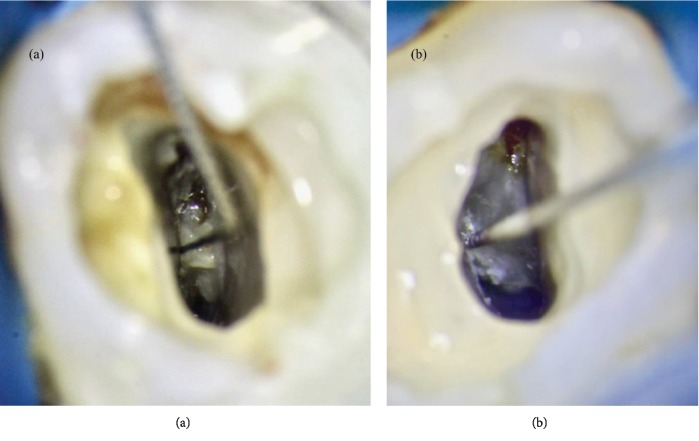
(a) First case: the canal portion between the buccal and the palatal poles was shaped using a bended sonofile #15 K. (b) Second case: the isthmus between P and D canals was shaped using a bended sonofile #15 K.

Therefore, in the first case report, the canal portion between the buccal and the palatal pole was shaped using a bended sonofile #15 K, as well as the isthmus between P and D canals in the second case report (Figure 7). For the same reason, in the second case, the MB and D canals were shaped in a very conservative way, using a ProFile 25/04.

**Figure 8:**
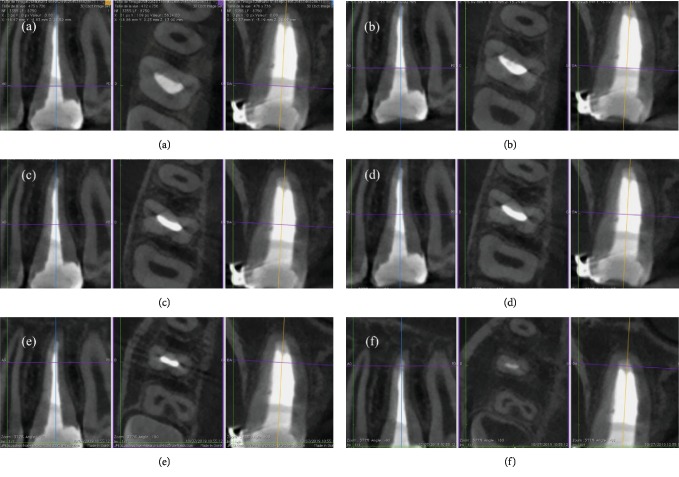
(a–f) CT images of the tooth at different level display, without any doubt, the C1 configuration of this maxillary right molar.

**Figure 9:**
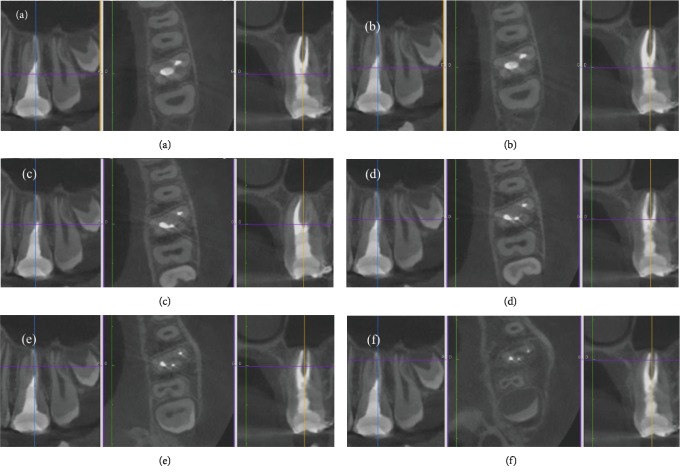
(a–f) CT images of the tooth at different level display, without any doubt, the C3 configuration of this maxillary left molar.

Moreover, the efficacy of irrigation should be enhanced in order to improve the chemical digestion, cleaning, and disinfection of the irrigants in these irregular canal areas. Indeed, ultrasonic activation of the irrigants, in case of straight canals, and sonic irrigation in case of thin and/or curved canals should be used. These procedures allowed to obtain a tridimensional cleaning without enlarging the apical foramen and the root canal taper. The last goal was to obtain a tridimensional filling. Obviously, two different filling techniques were used to manage these two maxillary first molars. For the first case, the unique option to fill in a tridimensional way this oval canal with a very large buncolingual diameter was to use a bioceramic material, like MTA Biorep. On the contrary, the option chosen to fill the intricate root canal network of the second case report was to use the carried based technique. Five months later unawares, the young patient came back to the Clinic of Dental Surgery of Dental School, University of Strasbourg, to take a CBCT in order to plane the surgical extraction of third molars. Based on an examination of the CBCT, we could visualize the exact configuration of these maxillary first molars. Therefore, the first case reported in this paper is, without any doubt, a rare case of a maxillary first molar with one root canal and C-shaped morphology, a C1 type according to Fan's classification (Figure 8), whereas the second case is probably the first case documented of a maxillary first molar with a C-shaped root canal and C-shaped root with complete fusion of the three roots, having a C3 configuration (Figure 9). It presented one independent oval MB canal and one C-shaped root canal that apically splits into two canals, whereas the cases reported by Moor presented a C-shaped root canal for the fusion of the D and P canals without the fusion of the three roots.

In general, tooth morphology is bilaterally symmetrical. According to Sabala et al., when present on one side, a C-shaped canal may be found in the contralateral tooth in over 70% of individuals [21]. Nevertheless, this convincement, that anatomical variations appear frequently contralaterally, can be partially confirmed in the present cases. Indeed, if it is true that the two first maxillary molars have both a C-shaped anatomy, it is equally true that the right maxillary first molar presents a C-shaped anatomy with only one oval root canal and the left maxillary first molar presents a C-shaped anatomy with a deep pulp chamber floor and three root canals.

**Figure 10:**
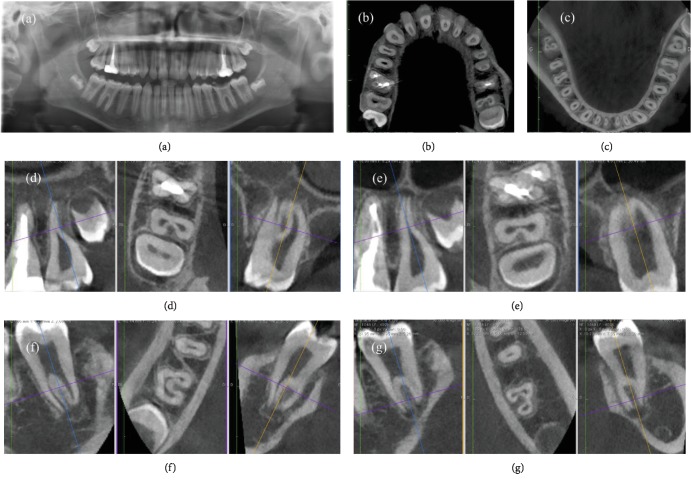
(a) Panorex; (b) CT-image of maxillary arch; (c) CT-image of mandibular arch; (d) CT images of the maxillary right second molar showing the C-shaped anatomy; (e) CT images of the maxillary left second molar showing the C-shaped anatomy; (f) CT images of the mandibular right second molar showing the C-shaped anatomy; (g) CT images of the maxillary left second molar showing the C-shaped anatomy.

Moreover, it was possible to highlight the C-shaped root morphology on both mandibular and maxillary second molars too (Figure 10). No doubt, reports of cases with unusual root and root canal anatomy have an important didactic value. Their documentation in case reports may facilitate the identification and successful management of equivalent cases, if they need an endodontic treatment.

## 4. Conclusion

Even if a maxillary first molar is considered a four-canal tooth, the clinician should be aware of the possibility of the presence of fewer canals with a C-shaped root canal configuration.

Therefore, the cases reported showed that the C-shaped canal system is an anatomic variation also occurring in maxillary first molars.

If conventional radiograph in these cases of C-shaped root canal for some clinicians is not clear enough, it would be better to take intraoperative CBCT, only if needed, especially on young patients.

Indeed, as these cases show, also in the case of C-shaped root canal configuration, the clinician's knowledge of the endodontic anatomy, a painstaking preaccess analysis of the preoperative radiographs, the systematic identification of the CEJ, using a periodontal probe, and the use of a surgical microscope coupled with the use of specific endodontic ultrasonic tips allow a high accuracy of the access cavity preparation and canal localization.
